# Spatial distribution of tuberculosis in a municipality in the interior of São Paulo, 2008-2013**^1^**

**DOI:** 10.1590/1518-8345.1064.2888

**Published:** 2017-06-05

**Authors:** Alcione Pereira Biffi Fusco, Ricardo Alexandre Arcêncio, Mellina Yamamura, Pedro Fredemir Palha, Amanda Alessandra dos Reis, Tatiana Ferraz de Araújo Alecrim, Simone Teresinha Protti

**Affiliations:** 2 Master's student, Programa de Pós-Graduação em Enfermagem, Universidade Federal de São Carlos, São Carlos, SP, Brazil.; 3 PhD, Associate Professor, Escola de Enfermagem de Ribeirão Preto, Universidade de São Paulo, PAHO/WHO Collaborating Centre for Nursing Research Development, Ribeirão Preto, SP, Brazil.; 4 Post-doctoral fellow, Inter-institutions Doctoral Program in Nursing, Escola de Enfermagem, Universidade de São Paulo, São Paulo, SP, Brazil and Escola de Enfermagem de Ribeirão Preto, Universidade de São Paulo, PAHO/WHO Collaborating Centre for Nursing Research Development, Ribeirão Preto, SP, Brazil.; 5 PhD, Adjunct Professor, Departamento de Enfermagem, Universidade Federal de São Carlos, São Carlos, SP, Brazil.

**Keywords:** Primary Health Care, Tuberculosis, Geographic Information Systems, Residence Characteristics, Incidence, Health Information Systems

## Abstract

**Objective:**

to describe the epidemiological clinical profile of tuberculosis and analyze the spatial distribution of cases in a municipality in the state of São Paulo.

**Method:**

descriptive and ecological study of cases of tuberculosis through the records in an information system. Descriptive statistics were used to calculate central tendency (mean) and chi-square test, with Yates correction or Fisher exact test, when necessary. The Kernel technique was also used to calculate the occurrence densities of tuberculosis cases, using a radius of 1000 meters. Type I error was set at 5%.

**Results:**

299 cases of tuberculosis were identified, with 290 (96.98%) being geocoded. The majority of these were male (n = 212; 70.91%), median age 40 years, and the pulmonary clinical form was predominant (n = 244, 81.60%). The distribution occurred in a non-random manner, observing important areas of the municipality with a higher density of cases of the disease.

**Conclusion:**

the study evidenced an epidemiological profile of tuberculosis cases similar to those in the literature; however, their distribution does not occur in a random manner, pointing to specific population groups that require greater management and planning of health services for the control of tuberculosis.

## Introduction

Tuberculosis (TB) has been present in mankind for about 8,000 years^1^ being considered as a serious but curable disease. It is estimated that in 2014 around 9.6 million TB cases occurred worldwide, with 6 million new cases and Brazil ranks 16th in terms of incidence within the 22 countries that account for 80% of the world TB burden, with a mortality rate of 2.6 deaths per 100,000 inhabitants, excluding the positive cases of Human Immunodeficiency Virus (HIV)^2^.

In the state of São Paulo, in the year 2013, the incidence rate was 38.26 per 100,000 inhabitants and the mortality rate was 2.05 per 100,000 inhabitants. In the same year, in the municipality of São Carlos, site of this study, the incidence coefficient of the disease was 24.52 per 100,000 inhabitants and the mortality rate of 2.53 per 100,000 inhabitants^3^.

Considering the worrisome epidemiological scenario of TB, in 2014 the World Health Organization (WHO), in the framework of the Sustainable Development Objectives, established the End TB strategy aiming to reduce 95% of mortality and 90% of TB incidence for the year 2035, using as baseline the 2015 indicators, as well as the elimination of the disease by 2050^2^. However, to reach these goals, there is a deadlock in the access and use of technologies that aid in the diagnosis and treatment of TB since in 2014 approximately three million people were unable to access health services worldwide^2^.

In this context, studies carried out in India and Brazil on the spatial distribution of TB indicate that in addition to the clinical characteristics of the individual, TB is also associated with different socioeconomic factors, such as income, schooling and household quality which must be considered in the definition of strategies for TB surveillance and control^4^.

The knowledge of the space is therefore relevant in the analysis of health and environmental relations, providing subsidies to management and services for control actions, which can cause significant changes in the morbidity and mortality of a society ^5^.

Through a review of the literature, using the TB and space and or environment descriptors, it was observed as a knowledge gap the non-use of geoprocessing tools to identify the priority areas for the control and surveillance of the disease in the region under study. For this reason it was proposed to characterize the clinical epidemiological profile of TB and to analyze the spatial distribution of the cases in a city of São Paulo state.

## Method

A descriptive and ecological study carried out in the city of São Carlos, located in the Center-East region of the state of São Paulo, Brazil, with an estimated population of 243,765 inhabitants in 2016, with socio-demographic characteristics of Human Development Index of 0.80 and Gini of 0.41. At the administrative level, the municipality has a geographical area delimited by five districts recognized as *Regional Health Administrations* (ARES - in Portuguese) that coordinate the health care network^6^.

In the municipality in question, care for patients with TB is centralized in the outpatient clinic for chronic infections, giving care and follow-up to patients with the HIV, TB, viral hepatitis and leprosy.

The study population consisted of all cases of TB residing in the municipality and registered in the TB-WEB information system from January 1, 2008 to December 31, 2013. Data collection occurred from October to December 2014. Duplicate cases, non-specific address, street dweller, non-existent address in the TB-WEB, residents outside the urban network and cases in which the outcome was a change of diagnosis were excluded.

The variables selected for the study were: socio-demographic profile (age, sex and schooling) and clinical-epidemiological profile (number of days between date of onset of symptoms and date of diagnosis of the disease, admittance type, clinical form of the disease, HIV co-infection, positive sputum examination, Purified Protein Derivative (PPD) test, endpoint status, treatment form, type of discovery and directly observed treatment (DOT) unit.

Initially, with the aid of the *Statistica* 10.1(r) software, we carried out absolute and relative frequency analyzes. For the continuous quantitative variables age and interval in days between date of onset of symptoms and date of diagnosis of TB, mean, median, minimum and maximum values, standard deviation and amplitude were calculated. Subsequently, the condition of endpoint (cure, quitting and death) was considered as dependent variable and the other variables as independent for performing univariate analysis, applying the chi-square test of proportions, with Yates correction, or exact test of Fisher, when necessary, setting the probability of type I error by 5%. It is important to note that any ignored or unfilled records were excluded from this step.

When the spatial analysis was used, the standardization and equalization of the addresses of the cases of residents in the urban area of the municipality were used from the StreetBase Basic(r) digital street base of the company Image(r), in *shape file* in projection WGS1984 - UTM - Zone 23S . Subsequently, the geocoding of the cases was started according to the terrain base using the TerraView software (version 4.2.2), generating a map embedded in a *Geographic Information System* environment. In this way, the geocoding was obtained by linear interpolation of the complete address, including the postal address code to a point in the corresponding street path between two points that define the numbering range of that street segments events.

In the sequence, the exploratory analysis of the spatial behavior of the events was carried out by Kernel point density, estimating the intensity of the point process throughout the study region, which allowed to highlight areas with higher case densities, that is, an exploratory surface interpolation, where it was possible to identify the "hot spots", which indicate the occurrence of clusters^7^. Considering a radius of 1000m, the thematic map of the distribution of cases of TB according to residence address was generated in ArcGis 10.2 software.

This research was approved under the nº 483.596, by the Research Ethics Committee of the Federal University of São Carlos.

## Results

Between 2008 and 2013, 315 cases of TB were reported and 16 cases were disregarded during the investigation due to a change in diagnosis, resulting in a total number of 299 cases of TB, the majority being male (n= 212; 70,90%), with a mean of 41.69 years, median of 39 years, minimum age of one and maximum of 90 years and standard deviation of 17.60 years. Still referring to the socio-demographic profile, 121 (40.46%) of the cases had education of four to seven years of study.

Regarding the clinical epidemiological profile, the mean interval in days between the date of symptoms and the date of diagnosis was 70.80 days and a median of 31 days, with a minimum time of one and a maximum of 1008 days, with standard deviation of 115.28 days. Table 1 shows the data referring to the clinical profile of the disease, with the majority of cases having an interval between 1 and 30 days of diagnosis (n = 143; 47,82%), was a new case (n= 240; 80,27%), pulmonary clinical form (n = 244; 81,60%) did not have HIV (n= 238; 79,60%).

**Table 1:** Clinical-epidemiological characteristics of Tuberculosis cases. São Carlos, SP, Brazil (2008-2013)

Variable		N		%
Interval in days between date of symptoms and date of diagnosis				
	1 to 30 days	143		47,83
	31 to 90 days	82		27,43
	91 to 180 days	49		16,39
	181 to 365 days	19		6,35
	Over 365 days	6		2,00
Admittance type				
	New case	240		80,27
	Recurrence	35		11,70
	Re-entry after quitting	23		7,70
	Re-treatment in failed cases	1		0,33
Clinical form				
	Pulmonary Tuberculosis	244		81,60
	Extra pulmonary tuberculosis	55		18,40
HIV* Coinfection				
	Yes	42		14,05
	No	238		79,60
	Ignored	19		6,35
Performing the Sputum Exam				
	Yes	147		49,16
	No	81		27,10
	Ignored	71		23,74
PPD**^†^**				
	Yes	17		5,69
	No	17		5,69
	Ignored	265		88,62
Closing situation				
	Cure	232		77,60
	Dropout	37		12,38
	Tuberculosis death	12		4,01
	Non-tuberculosis death	12		4,01
	Treatment failure	2		0,67
	Transfer	3		1,00
	Ignored	1		0,33
Form of treatment				
	Supervised	262		87,63
	Self-administered	34		11,37
	Not started	1		0,33
	Ignored	2		0,67
Type of discovery				
	Outpatient demand	195		65,22
	Urgency / emergency	31		10,37
	Diagnostic elucidation during hospitalization	63		21,07
	Contact research	1		0,33
	Active search in institutions	2		0,67
	Active Search in Community	1		0,33
	Discovery after death	2		0,67
	Continuity of treatment	2		0,67
	Ignored	2		0,67
Directly observed treatment unit				
	Hospital unit	15		5,02
	Basic health Unit	65		21,74
	Family health unit	27		9,03
	Tuberculosis control program	169		56,53
	Private clinic	2		0,66
	Self administered	6		2,00
	Ignored	14		4,69
	Other municipality	1		0,33

*Positive Human Immunodeficiency Virus

†Purified Protein Derivative Tuberculin Test

In the univariate analysis, according to Table 2, the variables age (p=0,0017) and education (p=0,0043) presented a statistically significant association with the treatment outcome.

**Table 2:** Endpoint situation and association of independent variables. São Carlos, SP, Brazil (2008-2013)

	Variables	Cure f(%)	Dropout f(%)	Death f(%)	p value
Age (n=281)					0,0017
	1 - 4 years	1 (0,36)	0 (0,00)	0 (0,00)	
	5 - 14 years	2 (0,71)	0 (0,00)	1 (0,36)	
	15 - 39 years	111(39,50)	28 (9,96)	1 (0,36)	
	40 - 59 years	84 (29,89)	7 (2,49)	6 (2,14)	
	60 years and over	34 (12,10)	2 (0,71)	4 (1,42)	
Gender (n=281)					0,6947
	Male	161(57,30)	28 (9,96)	9 (3,20)	
	Female	71 (25,27)	9 (3,20)	3 (1,07)	
Interval in days between date symptoms and diagnosis date (n=204)			0,3497		
	1 - 30 days	46 (22,55)	8 (3,92)	2 (0,98)	
	31 - 60 days	41 (20,10)	7 (3,43)	2 (0,98)	
	61 - 180 days	65 (31,86)	7 (3,43)	2 (0,98)	
	181 - 365 days	11 (5,39)	5 (2,45)	2 (0,98)	
	Over 365 days	6 (2,94)	0 (0,00)	0 (0,00)	
Education (n= 261)			0,0043		
	None	15 (5,75)	1 (0,38)	2 (0,77)	
	1 - 3 years	32 (12,26)	4 (1,53)	1 (0,38)	
	4 - 7 years	79 (30,27)	24 (9,20)	5 (1,92)	
	8 - 11 years	75 (28,74)	4 (1,53)	0 (0,00)	
	12 - 14 years	13 (4,98)	0 (0,00)	0 (0,00)	
	Over 15 years	6 (2,30)	0 (0,00)	0 (0,00)	
Admittance type (n= 261)			0,7432		
	New case	188 72,03)	26 (9,96)	9 (3,45)	
	Relapse	30 (11,49)	3 (1,15)	2 (0,77)	
	Re-entry after dropout	2 (0,77)	1 (0,38)	0 (0,00)	
Clinical Form (n=261)			0,9770		
	Pulmonary	189(72,41)	33 (12,64)	9 (3,45)	
	Extrapulmonary	25 (9,58)	4 (1,53)	1 (0,38)	
CoinfectionHIV+ (n= 264)			0,2827		
	Yes	27 (10,23)	6 (2,27)	0 (0,00)	
	No	198(75,00)	25 (9,47)	8 (3,03)	
Positive sputum test (n= 216)			0,4425		
	Yes	115(53,24)	20 (9,26)	4 (1,85)	
	No	61 (28,24)	11 (5,09)	5 (2,31)	
PPD* (n= 31)			0,3852		
	Yes	13 (41,94)	1 (3,23)	0 (0,00)	
	No	14 (45,16)	3 (9,68)	0 (0,00)	
Form of treatment (n=279)			0,0548		
	Directly Observed Treatment	201(72,04)	37 (13,26)	9 (3,23)	
	Self-administered	30 (10,75)	0 (0,00)	2 (0,72)	
DOT**^†^** unit (n=268)			0,7592		
	Hospital unit	11 (4,10)	0 (0,00)	1 (0,37)	
	Basic health unit	51 (19,03)	7 (2,61)	2 (0,75)	
	Family health unit	24 (8,96)	3 (1,12)	0 (0,00)	
	Outpatient clinic of chronic infections	127(47,39)	25 (9,33)	8 (2,99)	
	Private clinic	2 (0,75)	0 (0,00)	0 (0,00)	
	Self-administered	5 (1,87)	0 (0,00)	1 (0,37)	
	Other municipality	1 (0,37)	0 (0,00)	0 (0,00)	

*Purified Protein Derivative Tuberculin Test

†Directly Observed Treatment

**Figure 1:**
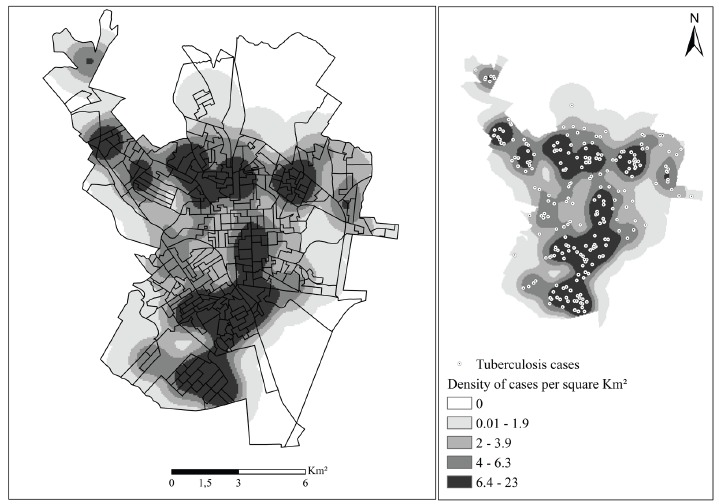
Map of the density of the distribution of TB cases in the municipality of São Carlos, SP, Brazil (2008-2013)

In the spatial analysis of the 299 reported cases, 290 (96.98%) were geocoded. The losses are due to failure to fill in the addresses, incorrect addresses and / or subnormal clusters (slums). By applying the Kernel technique, a non-random distribution of the disease could be observed (Figure 1), showing that the areas with the highest concentration of cases in the municipality occurred in the South and Southeast regions of the municipality.

## Discussion

This study aimed to characterize the clinical epidemiological profile of TB and to analyze the spatial distribution of cases in a São Paulo municipality. It was observed the predominance of males, in people in economically active age range, with less than eight years of education. These findings are in acordance with other studies carried out in other Brazilian scenarios^8^ ^-^ ^9^. Similarly, the study is consistent with the literature regarding the predominant clinical form of the disease, in which pulmonary TB was the most prevalent^2^ ^,^ ^7^ ^-^ ^8^.

Despite the predominance of cases of TB in young adults, it is noteworthy the existence of cases identified in childhood (One year old child with TB and one death due to TB in a 6 year old child). It is worth mentioning that the signs and symptoms of illness in childhood are nonspecific, making it difficult to diagnose the disease, from the asymptomatic to the severe ones, with signs of significant weight loss, complications and deaths^10^. The literature shows that when there is a child with TB, it probably acquired the disease due to frequent or long-term contact with an adult infected with *Micobacterium tuberculosis* ^10^.

Therefore, it is pointed out the need for careful investigation of the vaccination scar and scheme of BCG vaccine (Bacillus Calmette-Guérin) during the childcare visits until the sixth month of life, as well as to train health professionals for the care and clinical knowledge of signs and symptoms of TB in childhood^11^. These findings give rise to a discussion of the importance of systematic and integrated actions for the promotion, prevention and control of the disease, as an active search for early diagnosis, treatment, control of misconduct and control of communicants.

Although most cases were diagnosed by spontaneous demand at outpatient clinics (including primary health care), there was a significant amount in which the diagnosis was made only after hospital admissions (21,07%), which is probably due to the lack of early diagnosis in Primary Health Care (PHC) and even in the follow-up during DOT in the hospital environment, despite evidences of the cost effectiveness of PHC in the clinical management of TB patients^12^.

WHO has recommended PHC embedded on universal access systems, which are comprehensive. There is evidence that these arrangements are capable of covering 85% of the demands or needs of a territory^13^. Tax-financed public universal systems, organized from one territory, with PHC interacting with other health care points and with multiprofessional teamwork, had proven to be more effective and with lower-cost results for the improvement of health indicators in communities^13^.

The results also show that of the new cases, 77.60% were cured (below 85% recommended by the WHO) and 12.38% quitted treatment (above 5% of the goal tolerated by the WHO)^2^. Another identified deficiency was that 6.35% of the cases were not tested for HIV infection.

Regarding the association of the variables with the type of endpoint, it was verified that the dropouts occurred in people aged between 15 and 39 years, different from the cases of deaths, in which the age was between 40 and 59 years. Regarding education, both cases of dropouts and deaths presented years of study inferior to those that obtained the cure outcome. The results point to the relationship between TB and social determinants, showing that groups with greater social disadvantage tend to dropout from treatment frequently and are also more vulnerable to the development of multidrug resistance and deaths^14^ ^-^ ^15^.

From the spatial analysis, a pattern of distribution of the non-random disease was observed in the municipality investigated, denoting the formation of clusters of cases in areas supposedly at risk for the transmissibility of the disease and / or infection. It is important to highlight the largest cluster (dark area) in the South, which is classified as an area of high urban social vulnerability according to the classification criteria of the State System of Data Analysis Foundation^16^.

From the perspective of spatial distribution, the research integrated the health data available in the health information systems to environmental data, which made it possible to identify the distribution chain and the dynamics of TB in the municipality.

In the map of the distribution of TB cases in question, the highest density of cases in the South and Southeast regions, which are the most populated, shows an extreme variation of population characteristics, concentrating all types of urban occupation. Despite the constant economic growth of the region, it can be observed that these populations are in social vulnerability as demonstrated by Ferreira et al. (2012)^17^, these areas present precarious conditions of education, work and other services to the community.

Areas with severe deprivations in relation to decent housing and basic sanitation also tend to experience poorly resourced health services with limitations in terms of supply and services^7^. Although the Health Care Network in these areas has not been investigated, there are reasons to assume that there is a relationship between the occurrence of TB, the areas where people lived and the health systems and services present.

In the spatial analysis of TB, it is possible to visualize the geographical areas in which TB cases occurred and, therefore, need more attention, being it preventive or curative with the purpose of reorganizing health services to respond to the health needs of the population^7^.

The diagnosis and treatment of the disease in the city under study are centralized, which can lead to more structured actions (protocols, diagnostic inputs and medications) regarding case management and follow-up of these cases until they are cured, but a question emerges regarding the specific accessibility of these populations to health services.

Clinical management through PHC can contribute to a better quality of diagnosis and treatment, reduction of hospitalizations, proximity and strengthening of the link, and community trust with the user and health service, because PHC operates based on the its essential attributes i.e. longitudinality, comprehensiveness, focus on the family, coordination and orientation to the community^18^.

Perrechi e Ribeiro^19^ emphasize the importance of strengthening and restructuring TB control actions in PHC, prioritizing the search for respiratory symptomatology, control and monitoring of the disease, as well as the adequate and rigorous registration of Health Information Systems.

This work reinforces the need to organize TB care in the areas most affected by TB and hypothesizes that the organization and / or strengthening of PHC in these areas may favor early diagnosis, lower risk of transmission of the disease and quality of life of the families due to the fact that PHC is geographically close to the patients, their families and community, thus being able to intervene more quickly in the breakdown of the transmission chain.

In addition, it is worth mentioning that in order to achieve the goals set by WHO^2^, elimination of TB is not the sole responsibility of the TB Control Program, given the need for joint, integrated, decentralized and intersectoral action, since the early detection, treatment, follow-up and cure of TB are not the responsibility of a single specialty, but of all professionals and health workers from the perspective of Health Care Networks.

The study also calls attention to quality of records for an effective health surveillance system and thus supports a better decision-making process. During the survey of information in the TB-WEB information system, the compulsory notification form presented several blank or ignored items. This fact makes the system unreliable and does not fulfill its purpose, which is to give tools to the health teams in the provision and organization of health services. One study emphasized that surveillance strategies used to control TB should include reliable information systems and efficient methods for locating bacilliferous cases^9^.

Despite the evidence of the association of TB with social determinants^15^, the TB-WEB information system does not include data concerning the condition of housing, monthly income, source of income, support or social equipment used such as the family grant "Bolsa-Familia" (a government grant to vulnerable families), which would be important to measure the degree of social vulnerability of these TB patients and their families and their forces in the disease progression chain.

Regarding the limitations of the study, it is worth mentioning those related to the characteristic bias of ecological studies, making the findings of this investigation not suitable to be inferred casuistically for the individual level, being only representative for the populations. In addition, the acquisition of information through secondary data can bring errors inherent in the notification or typing of the data and possible biases in the investigation. Finally, individuals who have contracted the disease may have been infected in locations other than their home.

In a general way, the findings evidenced the TB problem in a municipality endemic in the state of São Paulo using geoprocessing resources for the first time as a tool to identify priority areas for disease control and surveillance. In addition, the study points out the difficulty of the context regarding progress towards the goal *End TB,* reinforcing the need for a social policy that favors the reduction of social inequities in health, strengthening PHC as an coordinator of Health Care Networks^13^.

## Conclusion 

The study evidenced a higher proportion of cases diagnosed for males with less than eight years of schooling in the pulmonary clinical form and with an estimated time to diagnosis after the onset of symptoms ranging from 1 to 30 Days. Among the new cases, the cure rate and the percentage of treatment dropout did not reach goals established by the WHO. Although the municipality is not considered a priority for disease control, TB has proved to be an endemic problem, concentrating on a non-random pattern in the South and Southeast regions, suggesting the spatial dependence of the disease on deficiencies related to the areas of quality living conditions, education and income. New investigations are necessary in order to confirm this hypothesis. 
